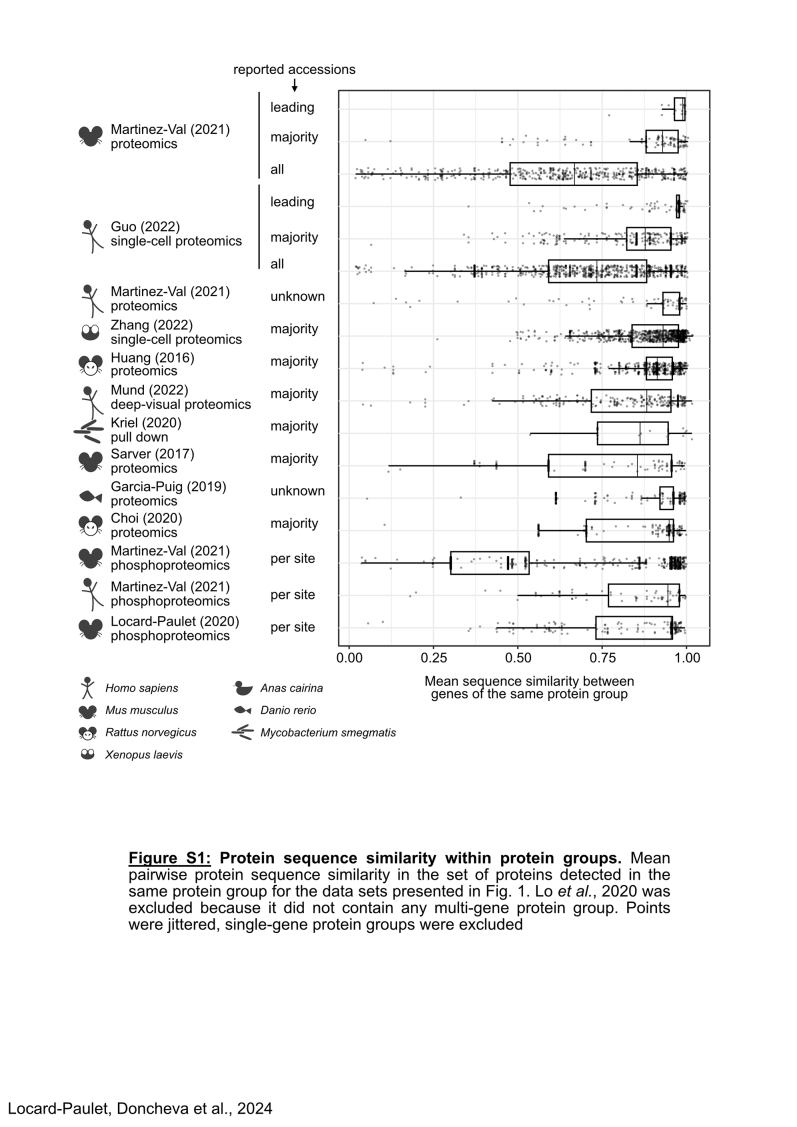# Functional Analysis of MS-Based Proteomics Data: From Protein Groups to Networks

**DOI:** 10.1016/j.mcpro.2024.100871

**Published:** 2024-10-31

**Authors:** Marie Locard-Paulet, Nadezhda T. Doncheva, John H. Morris, Lars Juhl Jensen

**Affiliations:** 1 Novo Nordisk Foundation Center for Protein Research, University of Copenhagen, Copenhagen, Denmark; 2 Institut de Pharmacologie et de Biologie Structurale (IPBS), Université de Toulouse, CNRS, Université Toulouse III-Paul Sabatier (UT3), Toulouse, France; 3 Infrastructure nationale de protéomique, ProFI, FR 2048, Toulouse, France; 4 Resource on Biocomputing, Visualization, and Informatics, University of California, San Francisco, California, USA

**Keywords:** Networks, Proteomics, Mass spectrometry, Bioinformatics, Biological databases, Protein groups, STRING, Cytoscape, Functional enrichment

## Abstract

Mass spectrometry-based proteomics allows the quantification of thousands of proteins, protein variants, and their modifications, in many biological samples. These are derived from the measurement of peptide relative quantities, and it is not always possible to distinguish proteins with similar sequences due to the absence of protein-specific peptides. In such cases, peptide signals are reported in protein groups that can correspond to several genes. Here, we show that multi-gene protein groups have a limited impact on GO-term enrichment, but selecting only one gene per group affects network analysis. We thus present the Cytoscape app Proteo Visualizer (https://apps.cytoscape.org/apps/ProteoVisualizer) that is designed for retrieving protein interaction networks from STRING using protein groups as input and thus allows visualization and network analysis of bottom-up MS-based proteomics data sets.

## Highlights

• Bottom-up proteomics data sets contain protein groups with multiple genes.
• Selecting one gene per protein group has an impact on functional analysis.
• Protein group representative genes need to be reported for GO term enrichment.
• Single-gene selection strongly impacts network visualization and analysis.
• Proteo Visualizer is a solution for STRING networks and protein groups in Cytoscape.

## Graphical Abstract

**Figure:**
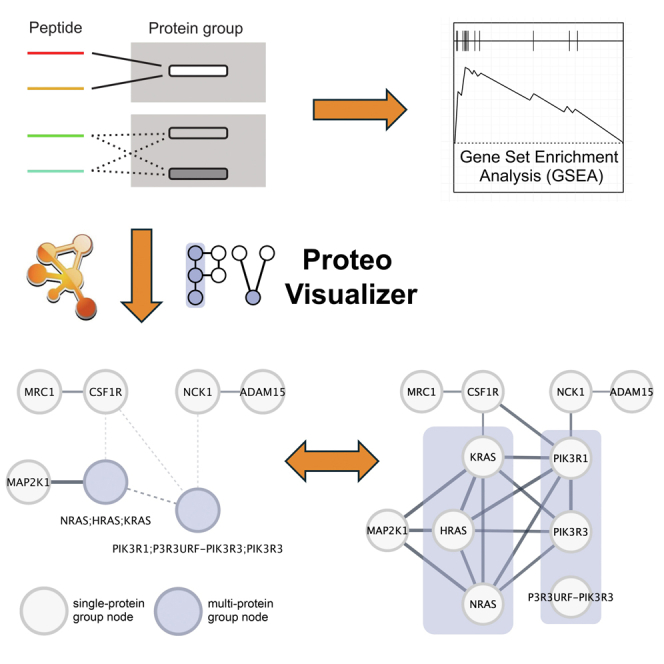

## In Brief

In bottom-up mass spectrometry, quantified protein groups can contain several genes. Based on 14 diverse proteomics datasets, we show that selecting only one gene to represent each group has little impact on GO-term enrichment, but authors should report what gene is selected for full transparency. We also show that single-gene selection strongly affects network analysis. Thus, we present the Cytoscape app Proteo Visualizer that retrieves STRING networks using protein groups as input to visualize and analyze MS-based proteomics data.

Nowadays, protein relative quantities can routinely be measured across samples using high-throughput mass spectrometry (MS). Classically, proteins are digested into peptides before MS analysis, often using trypsin for what is called bottom-up MS analysis, or shotgun proteomics when performed on a complex cell lysate (1, 2). Protein quantities are then inferred from their peptides’ MS signal (*i.e.*, protein inference) (3, 4). Not all peptides are equally detectable by MS, and enzymatic digestion can lead to loss of sequence coverage. Furthermore, aggregating peptide signals at the protein level remains a challenge, since peptides can be shared between proteins.

When one lacks peptide-level evidence to determine which proteins are present in a sample, it is usual to report protein groups that can be composed of one or several protein accessions. These gather all the protein accessions that cannot be distinguished based on the peptides detected in a given experiment. Usually, this is done using the parsimony rule (or Occam’s razor) that returns the minimal list of proteins sufficient to explain all observed peptides. Such an approach is used by default in most of the MS analysis tools where a single accession can only be in one protein group. Less frequently, all possible protein groups explained by the identified peptides can be listed in the final output, which can be useful for the specific analysis of proteoform variants, but results in accession redundancy that should be handled carefully for downstream functional analysis. Most software suites offer multiple options, allowing the users to choose the most suitable strategy for their data.

Regardless of how protein inference is performed, the nature of the protein groups obtained from a given shotgun experiment depends on the sample quality, the sensitivity of the experimental pipeline (and instrument), and the redundancy of the protein database used to match tandem-MS spectra to peptide sequences. Using well-curated proteomes such as the UniProt/SwissProt human proteome often results in only a few protein groups with more than one accession, whereas less-well-studied proteomes containing multiple proteins with similar sequences results in larger protein groups (where “larger” means more accessions per protein group). This is a bottleneck in the analysis of experiments where the input material is limited, such as single-cell proteomics. There is no agreement on how to handle protein groups for downstream analysis such as functional enrichment and protein–protein interaction networks. The methods sections of manuscripts very rarely describe how protein groups were handled for analysis that requires a single accession per feature. Most of the time a single accession per group is selected, but which accession and how it is selected is rarely reported. The impact of choosing one protein (or gene) over another on functional analysis is not known, making it difficult to assess what bias single-accession selection can generate on proteomics results.

Here, we investigate the protein groups identified in 14 high-throughput proteomics data sets. We show that different accessions in the same protein group usually have similar sequences but may not have the same functional Gene Ontology (GO) annotation(s). Our work also reveals that downstream functional analysis such as GO-term enrichment and biological network analysis can be impacted by which single protein accession is selected as input to the analysis. Although GO-term enrichment is quite robust when analyzing global proteomics data sets, the selection of a single gene in a protein group has a strong impact on network generation. Hence, we propose the new Cytoscape app Proteo Visualizer that allows the retrieval of STRING networks from a list of protein groups, their visualization, and functional analysis.

## Experimental Procedures

### Data Retrieval and Parsing

All the information about the data sets used in this study can be found in Supplemental Table S1. We worked with the Supplemental data associated with the publications, which were retrieved from the publisher websites or the associated ProteomXchange data repositories (5).

### Identifier Mapping: From UniProt Accession to Unique Gene Identifier

For each UniProtKB accession in the selected data sets, the corresponding protein sequence was retrieved from the UniProt Archive UniParc (6). Only in four of the data sets, there were a few missing sequences (1 in Guo human, 1 in Mund human, 5 in Martinez Val human, and 36 in Garcia-Puig zebrafish). For each species, all protein sequences were retrieved from STRING v11.5 (7) and used as a database for blasting the data set sequences using the following parameters: max_target_seqs=3, max_hsps=1, evalue=1e-2, and outfmt=6. For each UniProtKB accession, the best match(es) in STRING were identified as those with sequence identity > 90%. If more than one sequence fulfilled these criteria, we applied the following filtering criteria until only one sequence was left: max(identity), min(evalue), max(bit.score), min(Ensembl gene ID), where the Ensembl gene identifiers were extracted from the STRING aliases file for each species. See Supplemental Table S1 for a full overview of the identifier-matching numbers. For phosphoproteomics data sets (Martinez-Val *et al.*, 2021 (mouse phosphoproteomics (8)) and Locard-Paulet *et al.* (9)), we used the field “Proteins” of the site tables from MaxQuant. The other phosphoproteomic data set was analyzed with Spectronaut v14, and it was not clear what was reported in the field “PG.ProteinGroups” of the Supplemental Table S1.

### Gene Product Sequence Similarity

Homology scores, also referred to as *bit scores*, were retrieved from STRING v11.5 (7) for all pairs of genes in each of the species of interest. All missing bit scores were replaced by the minimum bit score for a given species (minimum pairwise similarity value). To correct for differences in sequence length, each gene pair bit score was divided by the minimum value of the two corresponding self bit scores (min(bit score gene1 *versus* gene1, bit score gene2 *versus* gene2)) to obtain self-normalized bit scores.

### Gene Functional Similarity

The GO-term annotations from STRING v11.5 were used to calculate the gene ontology similarity as follows. For each GO term *t*, its information content was calculated as *ic*(*t*) = −log(*p*(*t*)), where *p*(*t*) = freq(*t*)/freq(*root*) and freq(*t*) = ∣annot(*t*)∣ + ∑_*c*∈children(*t*)_ ∣annot(*c*)∣. The GO hierarchy was defined based on the Obo file used for STRING v11.5. Then, for each pair of proteins *i* and *j*, two measures were calculated as described by Jiang *et al*. (10). The remaining uncertainty ru and the missing information mi are defined as *ru*_*ij*_ = ∑_*t*_ *ic*(*t*) ⋅ 1 (*t* ∉ *P*_*i*_ ∧ *t* ∈ *P*_*j*_) and *mi*_*ij*_ = ∑_*t*_ *ic*(*t*) ⋅ **1** (*t* ∈ *P*_*i*_ ∧ *t* ∉ *P*_*j*_), where *P*_*i*_ and *P*_*j*_ are the sets of GO terms for proteins i and j, respectively. The overall normalized distance score is given by *s*_*ij*_ = sqrt(*ru*_*ij*_^2^ + *mi*_*ij*_^2^)/∑_*t*_ *ic*(*t*) ⋅ **1** (*t* ∈ *P*_*i*_ ∨ *t* ∈ *P*_*j*_). The functional similarity was reported as (1 − *s*_*ij*_).

### Gene Set Enrichment Analysis

All the GO-term analyses were performed with the GO-term annotation files from STRING v11.5 (7). We randomly selected one gene for each protein group (selection performed 10 times, see Supplemental Table S2 and the Zenodo repository for gene sampling tables and enrichment results), and used the GSEA function in R (package clusterProfiler, using the default option by = “fgsea”). We used two gene ranking strategies: by decreasing log2-transformed fold change, and by decreasing sign-logged *p*-value (−log10(*p*-value) with the sign of the log2(fold change), using decreasing absolute fold change for equal *p*-value). BH-adjusted *p*-values resulting from the GO-term enrichment were considered significant when ≤ 0.05. “2i” corresponds to the combination of two inhibitors as described in (11): CHIR99021 and Mirdametinib.

### Network Analysis

For each data set, STRING networks were retrieved for all gene identifiers using confidence cutoffs of 0.4, 0.7, and 0.9 for functional associations (*full STRING network*). In the resulting networks, genes belonging to the same protein group were annotated as such in order to determine the number of connections between genes in different protein groups. These numbers were used to calculate the probability of the protein groups being connected in a STRING network. Specifically, for protein group *a* and protein group *b*, we determined the number of edges *e*_*ab*_ that connect the two groups given a predefined STRING confidence cutoff. The size of each protein group was set to the numbers of protein group members *n*_*a*_ *= |a|* and *n*_*b*_ = *|b|*. The final probability of an edge to exist between the two groups is calculated as *p*_*ab*_ = *e*_*ab*_/(*n*_*a*_ ∗ *n*_*b*_).

### Proteo Visualizer App for Cytoscape

The app is implemented in Java under the BSD 2-Clause “Simplified” open source license (https://spdx.org/licenses/BSD-2-Clause.html) and the source code is hosted on github (https://github.com/scaramonche/ProteoVisualizer). The app is available for users at the Cytoscape app store: https://apps.cytoscape.org/apps/ProteoVisualizer.

Proteo Visualizer relies on the Cytoscape stringApp for retrieving networks from the STRING database (12) as well as the built-in Cytoscape CyGroups functionality for the creation and maintenance of the group nodes. In accordance with other Cytoscape apps, the main functionality is also available *via* commands and can be automatically executed from R or Python *via* the Cytoscape automation interface (13).

Currently, the app works as follows. Given a list of protein groups and user-defined settings for STRING (confidence cutoff, network type, and species), a network for all genes matched to the accessions is retrieved, keeping track of which nodes belong to which group. Then, genes that belong to more than one protein group are duplicated and the duplicated nodes are connected by an edge (with type *identity*). In the next step, protein group members are assigned to a *CyGroup*, a representative protein is chosen to be used for enrichment, and all groups are collapsed. Finally, all node and edge attributes are aggregated depending on their type and the size of the protein groups they belong to.

For the nodes that are part of collapsed protein groups, all numeric attributes such as COMPARTMENTS and TISSUES confidence scores are averaged over the members of each protein group. Selected textual attributes including the canonical UniProtKB identifier, display name, the list of known PDB structures, and developmental level and family information from Pharos are concatenated. In addition, whenever a protein group is collapsed, all edges connecting its members to other proteins or protein groups are replaced by one or multiple *meta* edges. The corresponding edge scores are summed up over all existing edges and divided by the number of all possible edges that could connect this protein group with another protein or protein group. For easy reference, the number of existing and possible edges between protein groups are saved as attributes in the Cytoscape Edge table and can be looked up there. The final STRING network might contain edges with confidence scores that are below the user-specified cutoff. Therefore, such edges are highlighted in the network view by using dashed edge lines.

Although a collapsed group node looks like any other STRING node, it can be uncollapsed, which leads to all its members and corresponding edges being displayed in the network view with a semi-transparent box behind them to indicate that they belong together (referred to as *compound node* visualization). In order to ensure compatibility with stringApp and general data import in Cytoscape, each protein group accession composition is stored in the *query term* column of the collapsed group node in the Node table, while each group member’s *query term* value is set to the accession used to retrieve it. The representative node for each group, which is used for enrichment analysis and network expansion with stringApp, is set by default to the first accession in the protein group but can be changed by the user later on.

Proteo Visualizer can also work with Omics Visualizer to combine the protein groups with time series or post-translational modification data sets. In such a case where protein group members can be assigned different information than the protein group, it is recommended to switch from the *compound node* visualization to *show group node* visualization of the expanded groups, which displays all group members and the group itself as separate nodes. The node attribute *protein group* can be used to keep track of which nodes belong together as being part of the same group.

## Results

### Data Sets Description

To evaluate the impact of protein groups on downstream functional analysis, we selected 14 publicly available data sets with different types of proteomics data (see Supplemental Table S1 for their full description). These were chosen to cover a large range of samples (bulk proteomics, phosphoproteomics, single-cell proteomics), sample preparation strategies (single injection or fractionated peptide samples, label-based or label-free quantification), instruments (a timsTOF from Bruker, and orbitrap instruments from ThermoFisher Scientific), and methods (including data-independent and data-dependent acquisition) (8, 9, 14, 15, 16, 17, 18, 19, 20, 21, 22, 23). All the analyzed samples are tryptic digests, but their depth, peptide coverage, and the quality of the seven species proteome annotation vary in ways that impact their protein group composition.

Throughout the manuscript, we call “protein groups” all the accessions or groups of accessions quantified in the data sets with the same set of peptides. It varies between studies whether protein groups include all possible accessions within each group or only the most probable protein(s) based on the entire set of detected peptides in a given sample. Many proteomics software tools offer multiple options. For two of the data sets (Martinez-Val *et al.*, 2021 mouse proteomics (8); and Guo *et al.*, 2022 (23)), we compared three strategies described in the largely used MaxQuant software, which report:

1. “Protein IDs”: All the identifiers of proteins contained in the protein group. They are sorted by decreasing number of identified peptides. In other words, these include all protein identifiers that match the set of peptides corresponding to the protein group.
2. “Majority Protein IDs”: Identifiers of those proteins that have at least half of the peptides that the leading protein group has (see below).
3. “Leading Proteins”: proteins containing the highest number of peptides in a given group. In case of a tie, multiple accessions are reported.

**Fig. 1:**
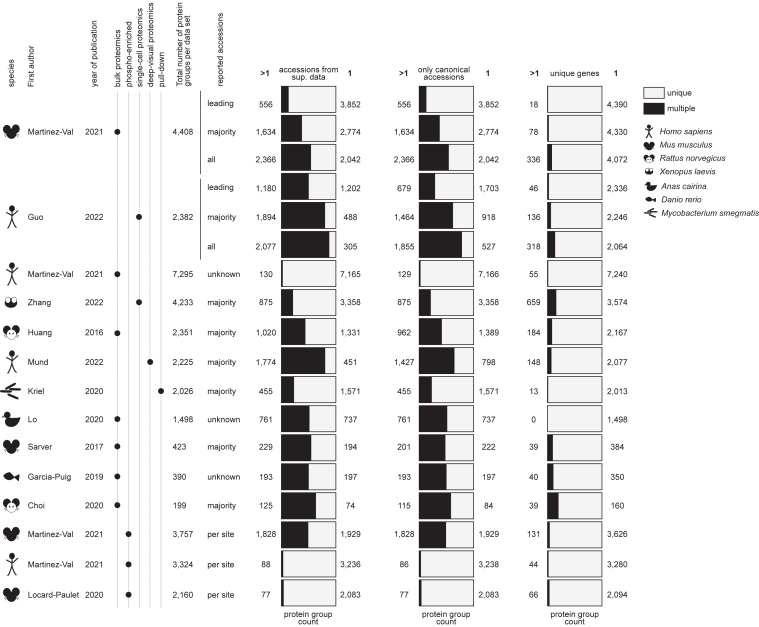
**Protein group composition of the data sets analyzed in this study.** Fourteen data sets were retrieved from the Supplemental data of published manuscripts. They cover seven species, all samples were trypsin digested and analyzed either as a whole in bulk or a single cell, or after selection of a subset of peptides (phosphorylated, associated to DNA, or belonging to segmented cell slide portions for deep-visual proteomics). Proportions and number of protein groups with one (“1”; *light grey*) or more than one (“>1”; *black*) accessions were reported as provided in the Supplemental Table S1 of the original publication (“accessions from sup. data”), after removing the isoform redundancy (“only canonical accessions”), and after matching to unique gene identifiers (“unique genes”). More information on the data sets is available in Supplemental Table S1.

For the other data sets analyzed with MaxQuant, we used the “Majority Protein IDs”, which are most commonly used in the literature. For the data sets analyzed with other software tools, we used the protein accession groups reported by the authors (Fig. 1). In phosphoproteomics data sets, output tables report one row per phosphorylated peptide with a sequence that may be found in several proteins, the accessions of which make up a protein group.

For each protein group retrieved from the Supplemental data, we matched the corresponding STRING gene identifiers based on sequence similarity to the reported accessions (see Experimental Procedures). The proportion of single- and multi-gene protein groups is very different depending on the data set and the accession reporting strategy (Fig. 1). For example, in Choi *et al.* (16), where the amount of material was limited (rat tendons), 19.6% of the protein groups matched more than one gene (multi-gene protein groups). As expected, this proportion was reduced in deep proteomes of well-studied organisms such as the mouse proteome of Martinez-Val *et al.* (8) where it was 1.8% (based on “Majority Protein IDs” in both cases). The duck liver proteome (Lo *et al.*) (20) did not contain any multi-gene protein group. It is thus absent from the next figures of this paper.

### Protein Sequence Similarity Within Protein Groups

**Fig. 2:**
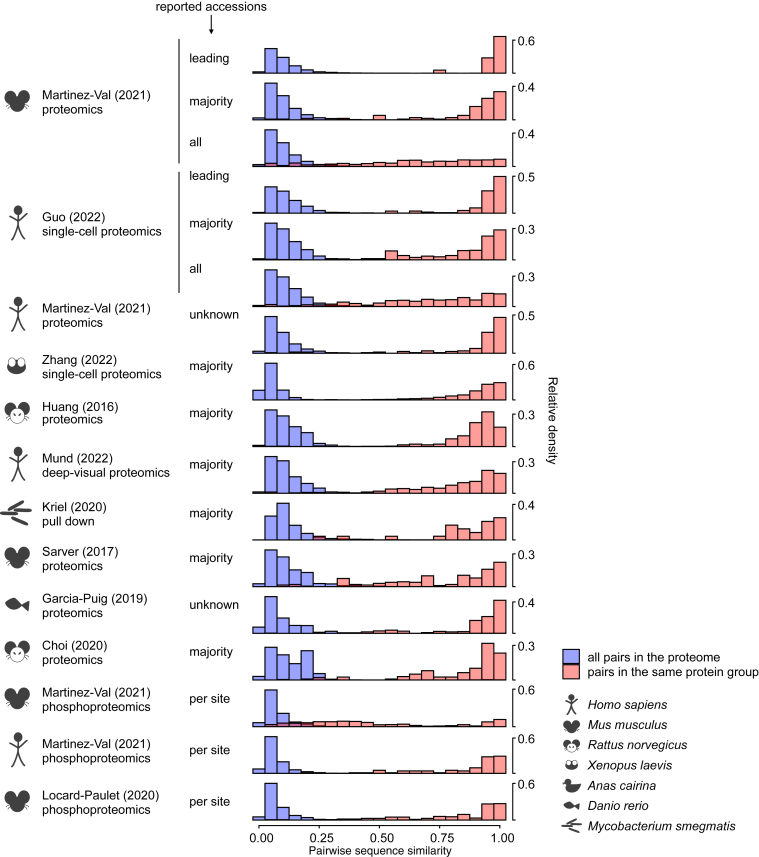
**Protein sequence similarity.** Distribution of pairwise protein sequence similarity in the set of proteins detected in each data set (*blue*) and in the pairs belonging to the same protein group (*red*) for the data sets presented in Fig. 1. Lo *et al.*, 2020 (20) was excluded because it did not contain any multi-gene protein group. Note that for greater legibility, the plots do not have the same vertical axis.

Genes within the same protein group share peptide sequences present in all of them, so we expect a high level of sequence similarity between them. Figure 2 presents the pairwise sequence similarity of all the gene pairs in the proteomes used for the 13 data sets containing multi-gene groups (blue bars). As expected, for the distribution of only the gene pairs that are in the same group, the sequence similarity increases (red bars), although some protein groups present a low mean sequence similarity (Supplemental Fig. S1).

The pairwise gene sequence similarity within protein groups increases when reporting “Majority Protein IDs” and “Leading Proteins” because these only contain the accessions matching the highest number of peptides. Conversely, in phospho-enriched data, the pairwise sequence similarity within groups decreases because groups are defined by one, or a very limited number of peptides containing a given phosphorylation site. Thus, the gene products belonging to the same protein groups are less similar, and this is amplified when using large proteome databases such as TrEMBL. The selected data sets contain two phosphoproteomics analyses of mouse samples: Martinez-Val *et al.* (mouse phosphoproteomics (8)) and Locard-Paulet *et al.* (9). In the first one, the authors used a fasta file that contained 43,539 sequences, including cleaved proteins and gene isoforms having some very similar sequences (UniProtKB/TrEMBL). Locard-Paulet *et al.* took a different approach since they used only the reference accessions of the UniProtKB/Swiss-Prot mouse database (16,699 sequences). This explains why the numbers of accessions per group are different between these two data sets (Fig. 1), as well as why the mean pairwise sequence similarity within protein groups differs (Supplemental Fig. S1).

Other factors can impact how similar gene sequences in the same groups are, such as the depth of the analysis, differences in sample preparation, and different filters applied to phospho-localization score, to name a few. These all influence the peptide group composition by impacting the number of unique peptides per group, which ultimately affects the presence of dissimilar gene products gathered in the same protein group.

### Functional Similarity Within Protein Groups

**Fig. 3:**
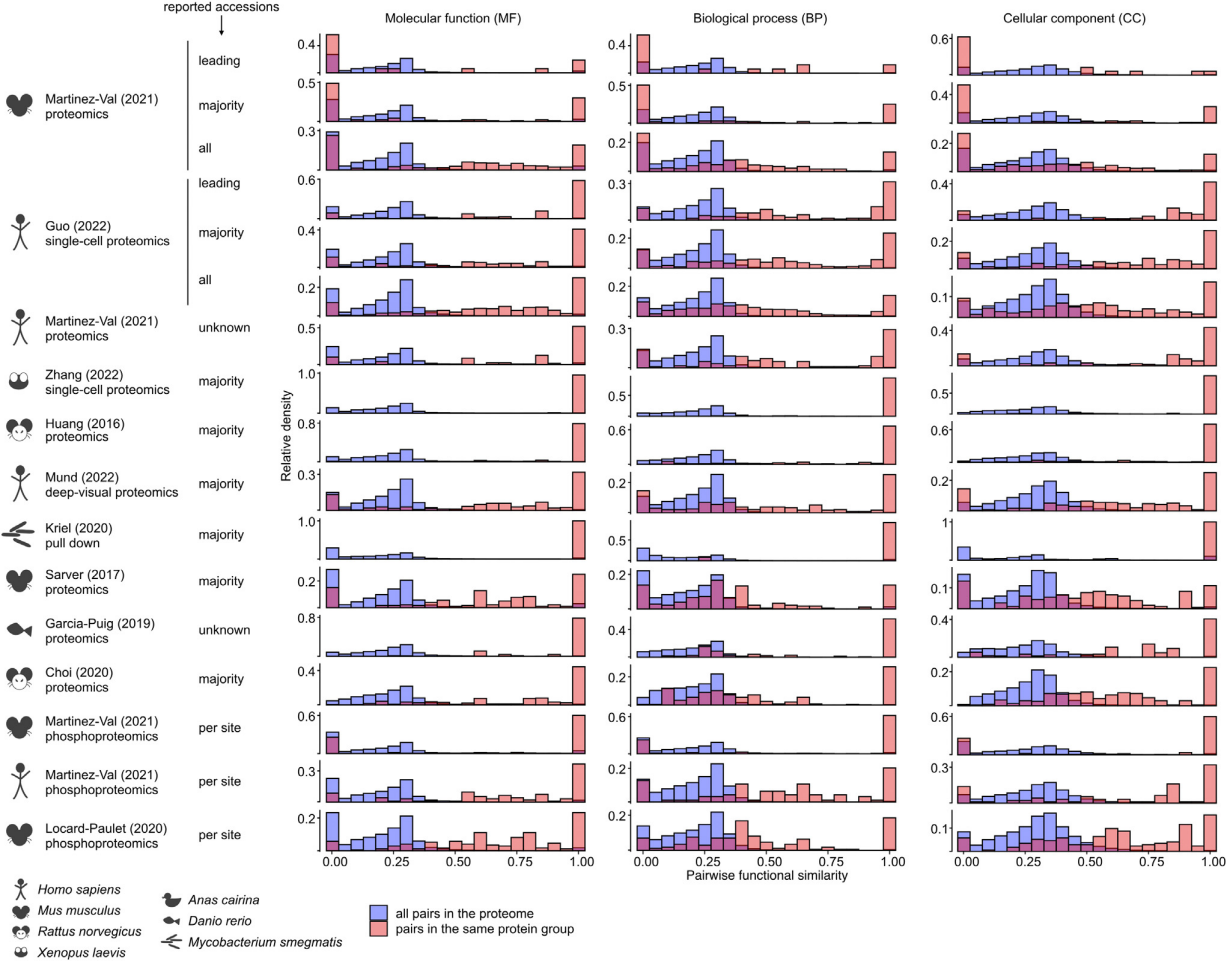
**Pairwise gene functional similarity.** Distribution of pairwise gene GO-term annotation similarity (*i.e.*, functional similarity) in the set of proteins detected in each data set (*blue*) and in the pairs belonging to the same protein group (*red*) for the three GO types (“MF” = molecular function; “BP” = biological process; “CC” = cellular component) in the data sets presented in Fig. 1. Lo *et al.*, 2020 (20) was excluded because it did not contain any multi-gene protein group. Two genes with no annotation have a functional similarity of 1. Note that for greater legibility, the plots do not have the same vertical axis.

Many resources exist that provide gene functional annotation coming from experimental evidence or prediction algorithms based on many parameters including sequence similarity, text mining, or co-expression (24, 25). They are often used when analyzing omics data sets, but cannot be directly mapped to protein groups. This raises several questions, such as how similar the annotated functions attributed to genes in the same group are, and if one can use the functional annotation of one gene to represent its protein group. We calculated pairwise GO-term annotation similarity (i.e. “functional similarity”) for all genes in the proteomes selected for the study (in blue in Fig. 3). Globally, pairwise functional similarity was slightly lower for molecular function (MF) than for cellular component (CC) and biological process (BP). For all data sets and GO types, gene pairwise functional similarities were shifted to higher values when focusing on gene pairs in the same protein group (red bars in Fig. 3), indicating more similar functional annotations. This can be due to functional annotations predicted based on sequence similarity since genes in the same group have higher sequence similarity (Fig. 2). Most of the genes in the same protein group have a functional similarity of 1, which corresponds to the exact same set of GO terms. It is worth noting that we considered a gene with no annotation as totally similar to another gene without annotation (functional similarity = 1) because two genes without annotation will have the same impact on GO-term enrichment analysis.

Figure 3 shows that pairwise functional similarity between genes of different proteomes depends on the species—which can be more or less annotated—and the redundancy in the database (TrEMBL *versus* UniProtKB/Swiss-Prot). Within pairs, the pairwise functional similarity is further impacted by the strategy chosen for protein group reporting (leading protein identifiers, majority protein identifiers, or all) and the deepness of the analysis (see Supplemental Fig. S2 for the mean pairwise functional similarity per protein group).

### Functional Analysis Using GO-Term Enrichment

GO-term enrichment is often performed to discuss proteomics results. It calculates the probability for a given GO term to be over-represented in a subset of regulated genes. GO-term annotation is available per gene, and there is no agreement on how to combine gene-level annotation with protein groups, so one has to select a single gene per protein group to perform the analysis. Manuscripts’ Experimental Procedures sections hardly ever describe how these single genes are selected, which lacks transparency and impedes reproducibility.

We observed that genes in the same protein group may not have the same functional annotation, which implies that selecting a single gene (or another) per protein group might have an impact on downstream functional analysis. To evaluate this, we performed gene-set enrichment analysis (GSEA) several times using different genes as protein-group representatives and compared the outputs. We worked with the proteomics data sets of Martinez-Val *et al.* (mouse proteomics, 2021 (8)) and Guo *et al.* (23) to evaluate the impact of protein group reporting on GO-term enrichment results. In both cases, we used comparisons from the original publications: the effect of 2-day treatment with Mek1/2 and Gsk3 inhibitors on the mouse embryonic stem cell proteomes (labeled “2i” based on Fig. 1 of Martinez-Val *et al.*; see Experimental Procedures for more information); the comparison of human oocyte proteomes after *in vivo* maturation (IVO) with the proteome of germinal vesicles (GV) for Guo *et al.*.

GSEA requires ranking the genes based on the amplitude of their regulation, or its significance. We ranked them by fold change or by signed log-transformed *p*-value (the sign corresponding to the direction of the regulation: up and down being positive and negative, respectively) when comparing two conditions. Each analysis was performed 10 times, randomly selecting one gene per protein group, and we calculated for each GO-term the proportion of draws for which it presented a GSEA *q*-value ≤0.05, which is very often used as the cutoff in the literature.

**Fig. 4:**
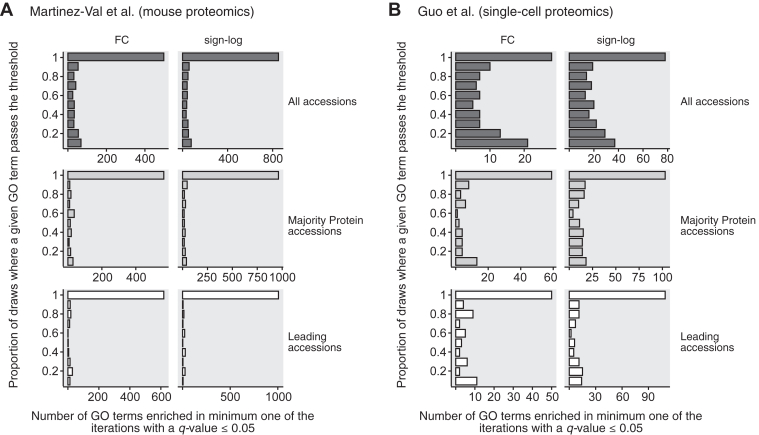
**Robustness of GSEA results depending on what gene is selected per protein group.** The proportion of times a given GO term is significantly enriched (*q*-value ≤0.05) after 10 random selections of single gene per protein group in the mouse proteomics data from Martinez-Val *et al.* (*A*) or the single-cell proteomics data set Guo *et al.* (*B*). All GO-term types were combined. We ranked the protein groups per decreasing log2-transformed fold change (“FC”) or signed log10(*p*-value) (“sign-log”) for GSEA analysis

Figure 4 shows the distribution of the proportion of iterations where each GO-term passed the enrichment threshold. Not all GO terms were enriched 100% of the time, so single-gene selection for protein groups has an impact on the GO-term enrichment results. Nevertheless, this impact is very mild in the classic use case of global proteomics using majority or leading protein accessions to report protein groups (Fig. 4*A*). In all cases, the proportion of times a GO-term is significantly enriched correlates with the significance of enrichment in each iteration and the median −log10(*q*-value) of all iterations (Supplemental Fig. S3*A*; results for majority protein accessions), which indicates that when considering the top regulated GO-terms, protein group single-gene selection has little impact.

Data sets with more multi-gene protein groups, such as the single-cell proteomics data from Guo *et al.* show less reproducibility (Fig. 4*B*), and in such cases, it is advisable to perform enrichment with several iterations of protein group single-gene selection to identify the GO-terms consistently enriched. In all cases, researchers should provide the list of single genes selected per protein group in such analysis for full transparency.

### Impact of Protein Grouping on Protein–Protein Interaction Networks

Like GO-term annotation, protein–protein interactions (or functional associations) are reported at the gene level in most if not all databases. It is common practice to use only one accession per protein group to generate a network. This has the obvious disadvantage that the resulting network only contains the single genes picked for each group: even if several genes have the same number of quantified shared peptides, only one will be present in the network and researchers may miss relevant nodes. Consequently, one may also miss relevant connections between nodes.

**Fig. 5:**
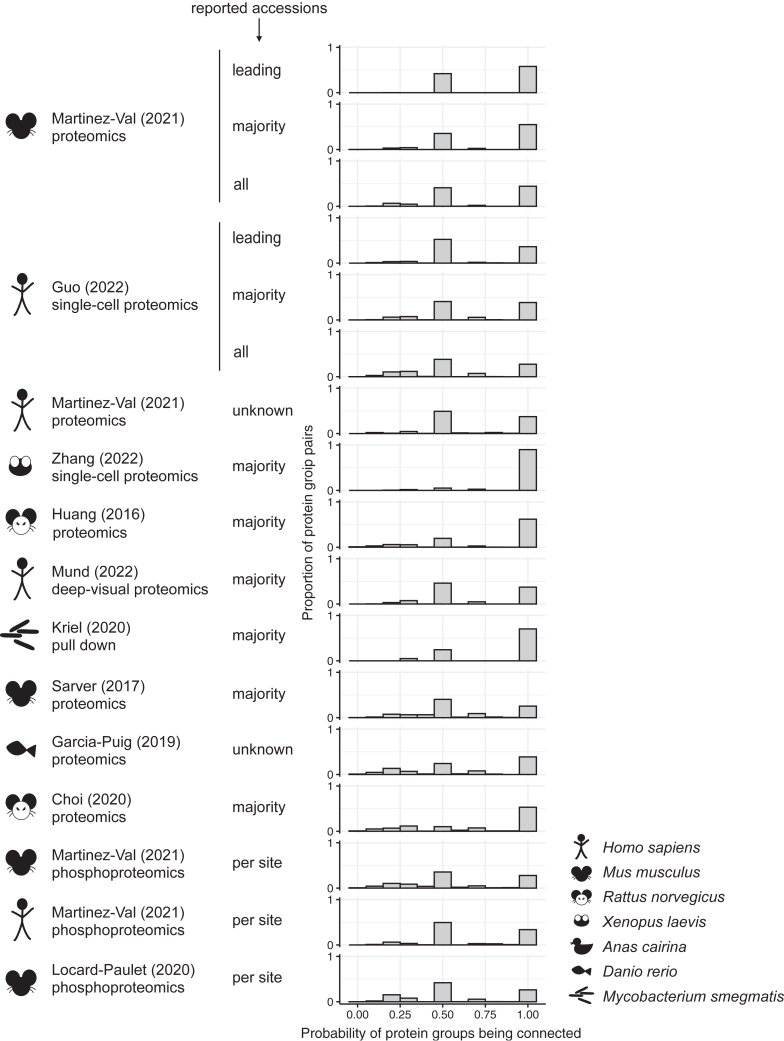
**Probability of protein groups being connected by an edge in a STRING network.** The proportion of protein group pairs with a given probability of being connected by high-confidence functional associations (score ≥0.7) is calculated for each data set. A probability of one means that two protein groups are always connected irrespective of which group member is chosen to represent the group, while a probability below one means that the connectivity depends on which member is picked.

We sought to estimate the impact of single-gene picking on the protein–protein interaction networks generated from MS-based proteomics data by retrieving functional associations from the STRING database (7). For each data set in this study, we calculated the probability for two protein groups to be connected in a network based on the number of STRING edges connecting each pair of genes from the groups divided by the number of all possible edges that could connect the two protein groups (Fig. 5). For completeness, we performed the analysis using three different confidence score cutoffs for functional associations from STRING, namely 0.4, 0.7, and 0.9 (see Supplemental Fig. S4 for scores 0.4 and 0.9).

Although many protein group pairs are always connected irrespective of which group member would be chosen to represent the group (probability peak at 1), the results consistently show that a large portion has 0.5 or less probability of appearing in a network created after the selection of one gene per protein group. Since the “probability of protein groups being connected” directly depends on the number of genes in each protein group, we observe distinct peaks at 0.5, 0.33, or 0.67. These values represent pairs of protein groups where one group has only one member and the other group has either two or three members, with at least one being connected with the first group and one not. Indeed, ∼90% of all pairs considered for the analysis (pairs of protein groups for which at least one protein group has more than one member) consist of a protein group with only one member and a protein group with more than one member. The appearance of distinct peaks is thus a consequence of counting interactions; these will not all have identical probabilities if we account for the varying probabilistic confidence scores of the underlying interactions from STRING. This is implemented in the Proteo Visualizer app described in detail later.

Overall, this network analysis indicates that single-gene selection has more impact on the network connectivity than on functional enrichment, and this is the case for any strategy chosen for protein grouping.

### Protein Group-Aware Network Analysis With Proteo Visualizer

**Fig. 6:**
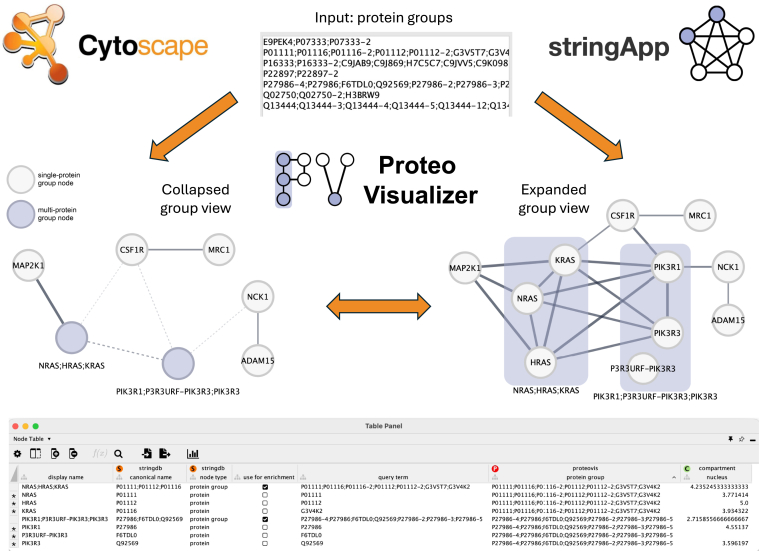
**Proteo Visualizer app overview.** The app input dialog allows users to provide the protein groups (shown here) as well as to set the STRING network retrieval options such as organism, network type, and confidence cutoff, which are needed for stringApp to retrieve the network. The two network representations show the same network with two multi-protein groups, which can be visualized either as collapsed (*left*) or expanded *compound* nodes (*right*). The visualized subset of nodes corresponds to regulated protein groups from Guo *et al.* (see Fig. 7). The Node table exemplifies some of the information available in Cytoscape *via* stringApp for each protein group and its members. The rows indicated with ∗ are only shown when the protein groups are expanded.

Based on the lack of consistency of how protein-group members are connected in a network as shown for STRING networks, we concluded that single-gene picking is not an option for building protein networks from bottom-up MS-based proteomics data. Thus, we developed a Cytoscape app named Proteo Visualizer, which allows the generation of networks with nodes that represent protein groups and are composed of several genes (Fig. 6). Although the current version only supports STRING networks and is optimized to seamlessly work with stringApp, future versions of the app will be agnostic to the source of the network and work on user-generated data such as affinity purification-mass spectrometry (AP-MS) or networks retrieved from IntAct (26) or GeneMania (27).

The main idea behind the Proteo Visualizer app is that users can provide a list of protein groups instead of single accessions as input to the network query. The app then uses this list to retrieve a STRING network and to create a collapsed group node for each protein group in the resulting network. Thereby, all node and edge attributes are automatically aggregated suitably depending on their type and meaning. The final network has the look and feel of any other STRING network and is compatible with stringApp’s functionality, in addition to a few extra features. In particular, users can uncollapse any group of their choice and explore the node information of the individual members, which shows up as separate rows in the Node table.

The node and edge attribute aggregation are designed such that group nodes and edges connecting such nodes represent an “average” of the information in the protein groups. For example, numeric protein group attributes (tissue and subcellular localization scores) are averaged over the values of all group members. In the case of numeric edge attributes (STRING evidence scores), the individual edge scores are summed up and divided by all possible edges that could connect the protein group members. This means that if an edge is missing, its score is assumed to be 0 and influences the final aggregated score of the protein group edge. As a result of this aggregation strategy, some protein group edges can have confidence scores that are lower than the confidence cutoff specified when retrieving the network and thus are represented as dashed edge lines.

In addition, the expanded group representation enables users to break down the averaged group-level edges into the individual interactions of the group members. For instance, in the network shown in Figure 6, we can distinguish two cases for the GTPases protein group *NRAS;HRAS;KRAS*: the Dual specificity mitogen-activated protein kinase kinase 1 (MAP2K1) is connected with all group members, whereas the Macrophage colony-stimulating factor 1 receptor (CSF1R) only interacts with KRAS from this protein group. NRAS, KRAS, and HRAS have a high sequence similarity and are involved in the same signaling pathway, but only KRAS is reported as part of the Reactome complex S-Farn-Me-PalmS KRAS in the CSF1 signaling pathway. This explains why CSF1R is only associated with KRAS (given a functional association score threshold of 0.7). If HRAS or NRAS were chosen as representative single genes for this protein group, this information would be missing from the network.

**Fig. 7:**
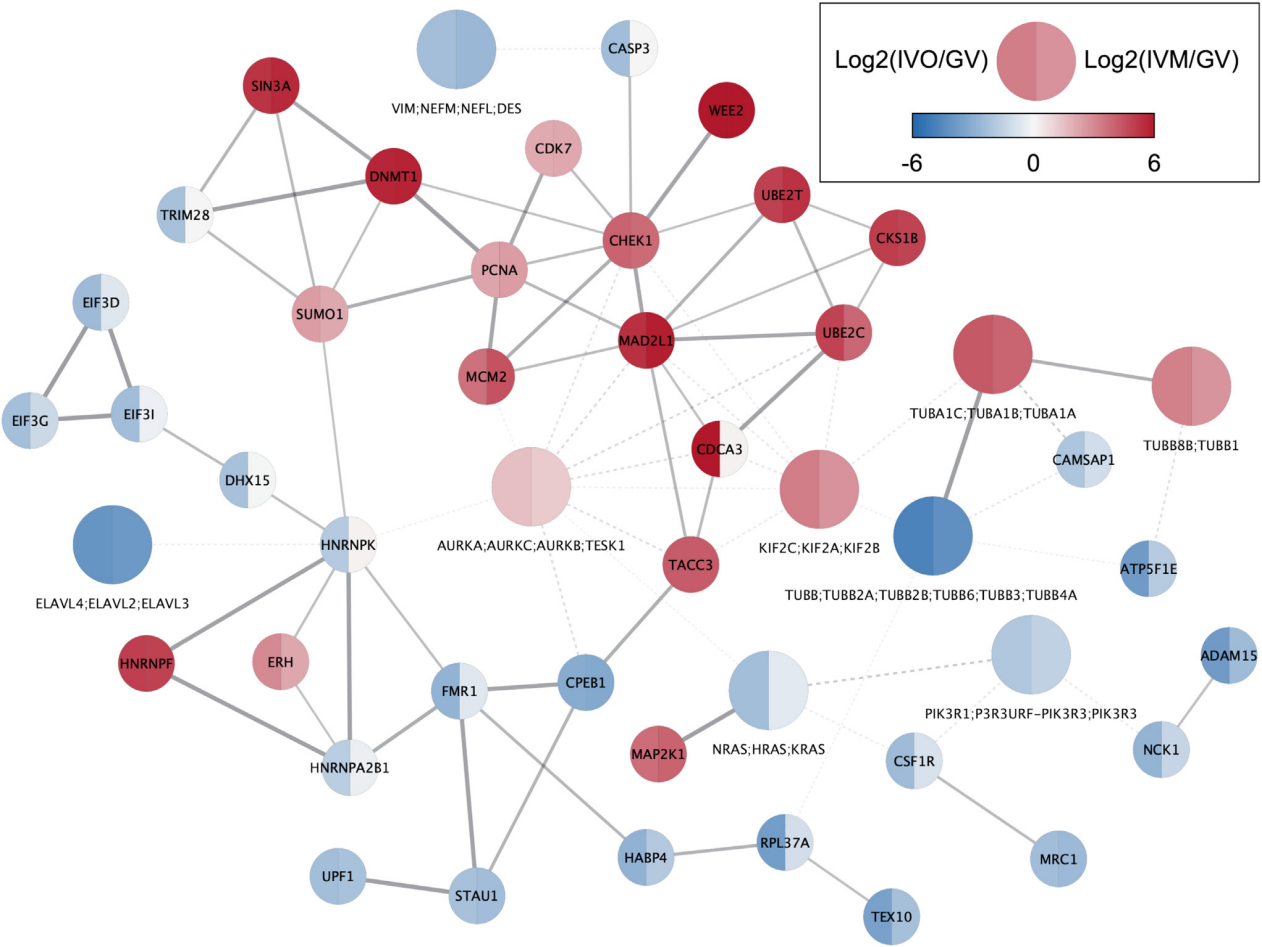
**Network visualization with Proteo Visualizer and Omics Visualizer.** A high-confidence STRING network (functional association confidence score ≥0.7) was retrieved with Proteo Visualizer using as input the set of regulated protein groups reported by Guo *et al.* (23) and used for the enrichment analysis in the previous section. Protein groups are shown as larger nodes and Omics Visualizer is used to map the log2-transformed fold change values of the comparison of human oocytes after in-vivo maturation (IVO) or in-vitro maturation (IVM) with germinal vesicles (GV) onto the nodes. Dashed edge lines indicate protein group edges that have lower aggregated confidence scores than the confidence cutoff specified when retrieving the network.

To make Proteo Visualizer networks compatible with the STRING visual style in Cytoscape as well as with performing enrichment analysis *via* stringApp, each group node is represented by one of the protein group members. By default, this is the first accession in the protein group, but the so-called group representative can be changed by the user later on. Furthermore, the Proteo Visualizer networks are compatible with any of the network clustering algorithms in clusterMaker2 (28) as well as other Cytoscape apps such as Omics Visualizer (29), thus allowing users to perform typical downstream analysis tasks while keeping the full resolution of the proteomic groups detected by MS-based proteomics data. This is exemplified in Figure 7, where we show the network of regulated protein groups from Guo *et al.* (23) together with the fold change values resulting from the comparison of human oocyte proteomes after *in vivo* maturation (IVO) and *in vitro* maturation (IVM) with the proteome of germinal vesicles (GV). A quick how-to guide describing the steps necessary to create such a visualization is available at https://jensenlab.org/training/proteovisualizer/.

## Discussion

MS-based proteomics results rarely reach single gene resolution, and researchers analyze quantities of protein groups instead of single gene products. From the analysis of 14 published proteomics data sets, we show that the presence and number of multi-gene protein groups depend on the type of sample and the set of protein sequences used for the MS search. Deep proteomics data sets only contain a few multi-gene protein groups, whereas single-cell proteomics, deep-visual proteomics, and phosphoproteomics data sets contain a high number of multi-gene protein groups. This impacts downstream analysis, and multi-gene protein groups need to be specifically handled when using gene annotation and building networks.

Although most genes in the same group have similar gene product sequences, their functional annotation can be different. This does not impact much reproducibility of GO-term enrichment when working with a reasonable number of multi-gene protein groups but can become an issue when the proportion of such groups increases. Since the gene selected for annotating the group impacts GO-term enrichment outputs, it is important to report what gene is selected to represent each group when publishing results. When working with data containing many multi-genes protein groups (for example when working with single-cell data or post-transcriptional modifications), we advise performing several GO-term enrichments after random selection of one gene per protein group and consider the GO terms that are reproducibly enriched as strong candidates.

In this work, we show that the selection of a single gene from each protein group has a strong impact on the network built from a given data set. Picking one gene per protein group can lead to two levels of information loss: 1) potential genes of interest are removed by keeping only one gene per group; 2) connections between nodes can be lost because all genes do not have the same level of protein-protein association annotation. Thus, we propose the Cytoscape app Proteo Visualizer, which allows the creation of STRING networks of protein groups and their visualization in Cytoscape. With this application, one can build networks that contain all the genes that can be present in a data set, grouping the ones that cannot be unambiguously distinguished based on their detected peptides. It is designed to easily visualize proteomics data containing many protein groups, such as single-cell proteomics data, and can be used in combination with other Cytoscape apps for downstream analysis.

## Data Availability

The Proteo Visualizer code is publicly available here: https://github.com/scaramonche/ProteoVisualizer. All the code associated with the manuscript figures and analysis are available here: https://zenodo.org/doi/10.5281/zenodo.10931932.

## Supplemental data

This article contains supplemental data.

## Conflict of interest

The authors declare that they have no conflicts of interest with the contents of this article.

## Figures and Tables

**Supplemental Fig. S4:**