# Supplementary material for: Functional Analysis of MS-Based Proteomics Data: From Protein Groups to Networks
Source: Mol Cell Proteomics. 2024 Oct 31;23(12):100871. doi: 10.1016/j.mcpro.2024.100871 (PMC11667155; doi:10.1016/j.mcpro.2024.100871)
Supplement: Supplemental Fig. S1 [file mmc3.pdf]

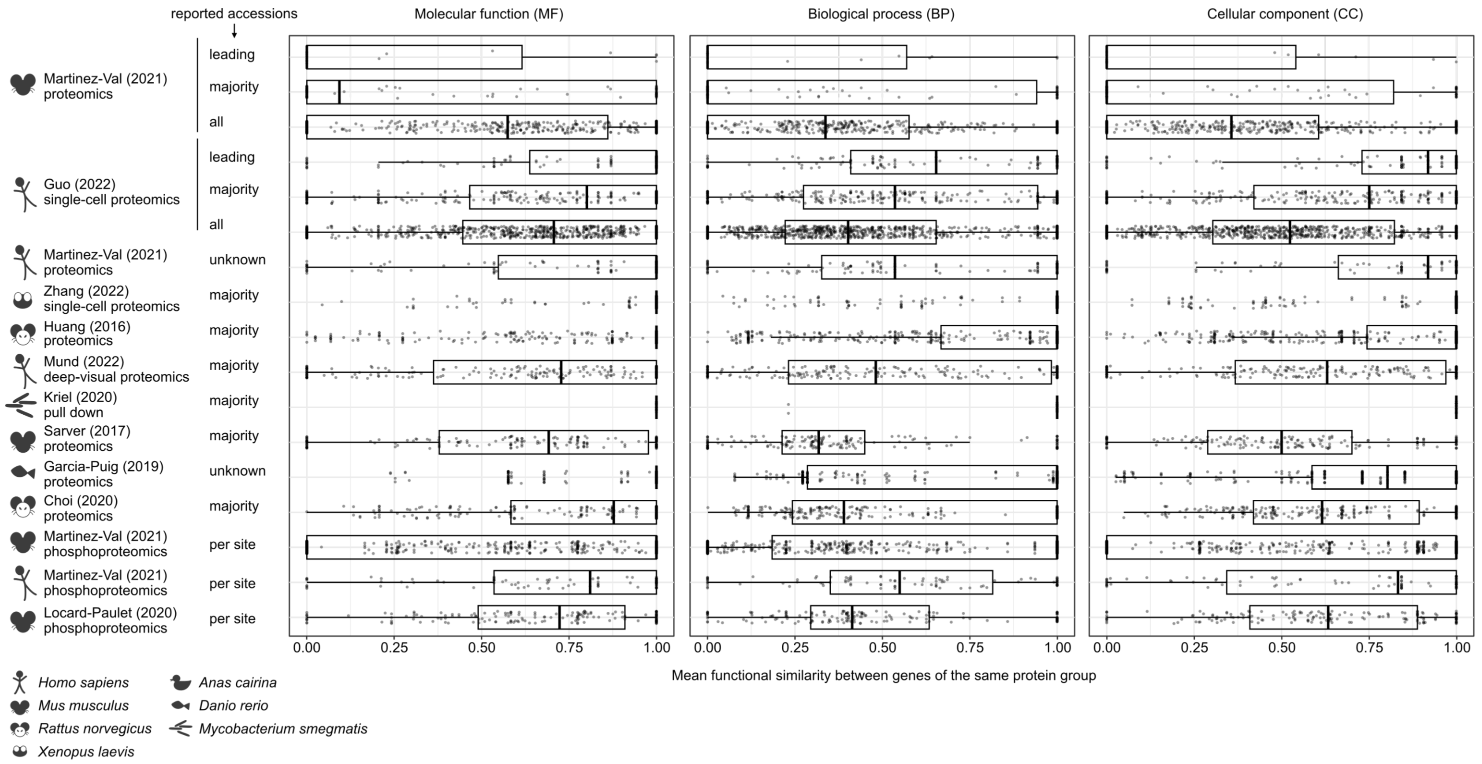

<u>Figure S2:</u> Pairwise gene functional similarity within protein groups. Mean pairwise gene GO-term annotation similarity (*i.e.* functional similarity) in the set of genes detected in the same protein group for the data sets presented in Fig. 1 (Lo *et al.*, 2020 (12) was excluded because it did not contain any multiple-gene protein group, single-gene protein groups were excluded), points were jittered. “MF” = molecular function; “BP” = biological process; “CC” = cellular component.
