# Supplementary material for: Functional Analysis of MS-Based Proteomics Data: From Protein Groups to Networks
Source: Mol Cell Proteomics. 2024 Oct 31;23(12):100871. doi: 10.1016/j.mcpro.2024.100871 (PMC11667155; doi:10.1016/j.mcpro.2024.100871)
Supplement: Supplemental Fig. S2 [file mmc4.pdf]

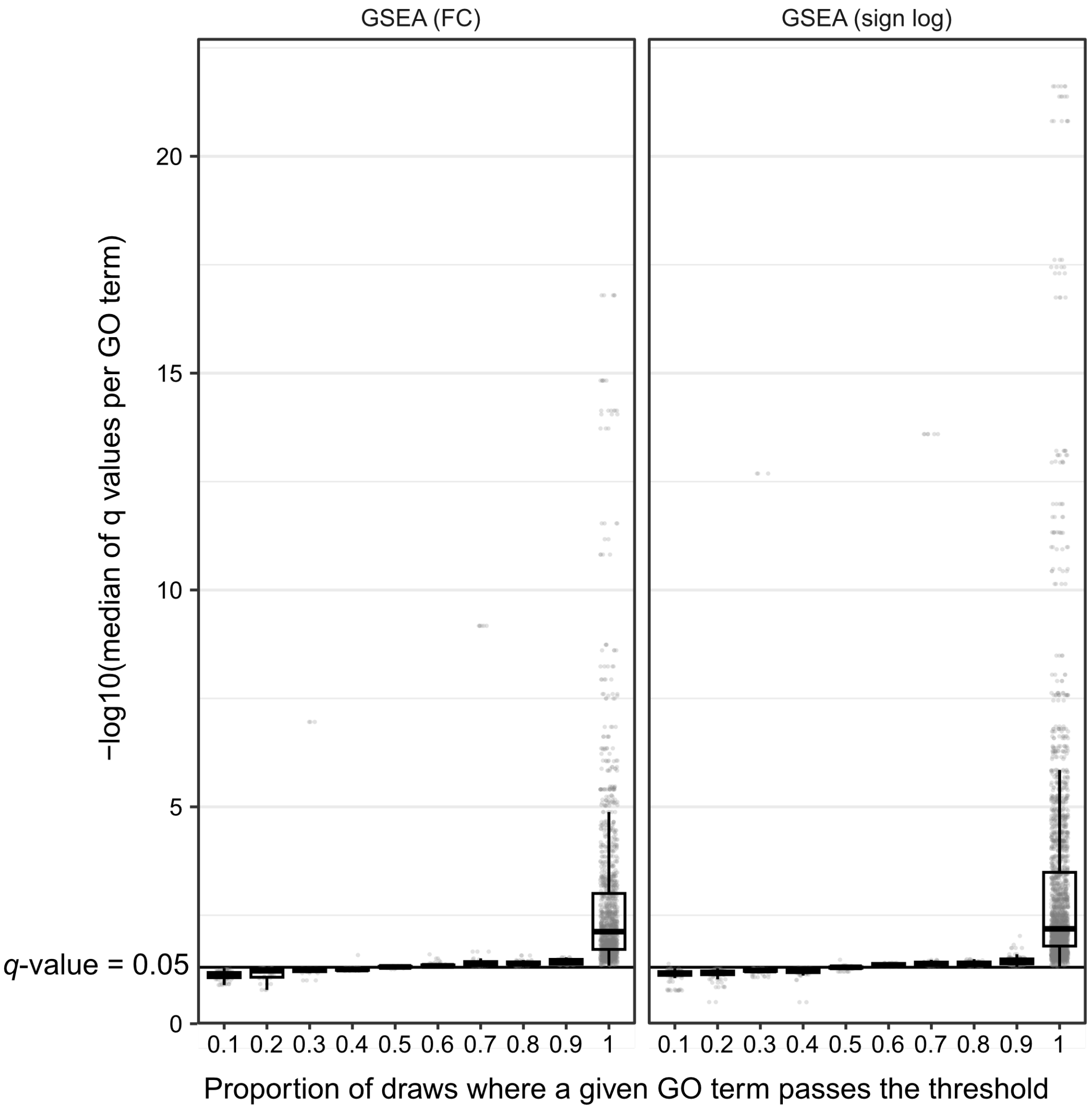

Figure S3: Enrichment reproducibility is correlated with GO-term *q*-values. Proportion of draws where a given GO term has a q-value ≤ 0.05 (horizontal axis) vs the -log10(median of q-values of all the draws) in the GSEA tests presented Figure 4. Points are jittered for visibility, and the q-value threshold of 0.05 is indicated by a horizontal line. For this figure, we only plotted the data reporting majority protein identifiers.
