# Supplementary material for: Functional Analysis of MS-Based Proteomics Data: From Protein Groups to Networks
Source: Mol Cell Proteomics. 2024 Oct 31;23(12):100871. doi: 10.1016/j.mcpro.2024.100871 (PMC11667155; doi:10.1016/j.mcpro.2024.100871)
Supplement: Supplemental Fig. S3 [file mmc5.pdf]

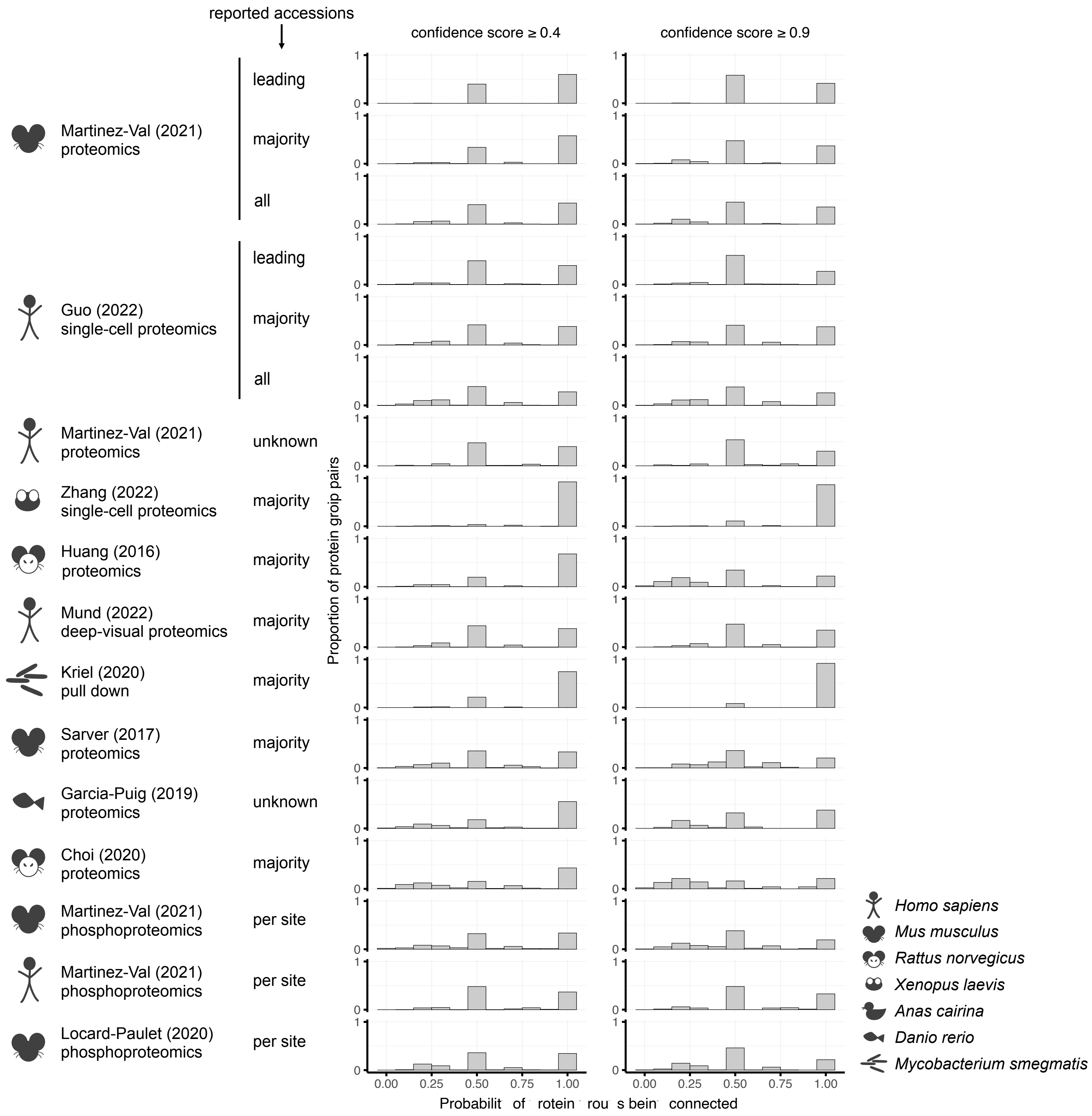

<u>Figure S4:</u> Probability of protein groups being connected by an edge in a STRING network. Proportion of protein group pairs with a given probability of being connected by functional associations (score ≥ 0.4 or ≥ 0.9) is calculated for each data set. A probability of 1 means that two protein groups are always connected irrespective of which group member is chosen to represent the group, while a probability below 1 means that the connectivity depends on which member is picked.
